# Association between bacterial community and cadmium distribution across Colombian cacao crops

**DOI:** 10.1128/spectrum.03363-23

**Published:** 2024-05-30

**Authors:** Claudia Jaramillo-Mazo, Daniel Bravo, Beatriz E. Guerra Sierra, Javier C. Alvarez

**Affiliations:** 1 Research Group in Biological Sciences and BioProcess (CIBIOP), School of Applied Sciences and Engineering, EAFIT University, Medellín, Colombia; 2 Laboratory of Soil Microbiology & Calorimetry, Corporación Colombiana de Investigación Agropecuaria AGROSAVIA, Mosquera, Colombia; 3 Research Group in Agro–Environmental Biotechnology and Health (MICROBIOTA), Facultad de Ciencias Exactas, Naturales y Agropecuarias, Universidad de Santander, Bucaramanga, Colombia; University of Torino, Torino, Italy

**Keywords:** bacterial community composition, pseudo-total soil Cd content, cacao-seed Cd content, soil physicochemical factors, linear mixed-effect model

## Abstract

Assessing the bacterial community composition across cacao crops is important to understand its potential role as a modulator of cadmium (Cd) translocation to plant tissues under field conditions; Cd mobility between soil and plants is a complex and multifactorial problem that cannot be captured only by experimentation. Although microbes have been shown to metabolize and drive the speciation of Cd under controlled conditions, regardless of the link between soil bacterial community (SBC) dynamics and Cd mobilization in the rhizosphere, only a few studies have addressed the relationship between soil bacterial community composition (SBCC) and Cd content in cacao seeds (Cd_seed_). Therefore, this study aimed to explore the association between SBCC and different factors influencing the distribution of Cd across cacao crop systems. This study comprised 225 samples collected across five farms, where we used an amplicon sequencing approach to characterize the bacterial community composition. The soil Cd concentration alone (Cd_soil_) was a poor predictor of Cd_seed_. Still, we found that this relationship was more apparent when the variation within farms was controlled, suggesting a role of heterogeneity within farms in modulating Cd translocation and, thus, seed Cd content. Our results provide evidence of the link between soil bacterial communities and the distribution of Cd across Colombian cacao crops, and highlight the importance of incorporating fine-spatial-scale studies to advance the understanding of factors driving Cd uptake and accumulation in cacao plants.

**IMPORTANCE:**

Cadmium (Cd) content in cacao crops is an issue that generates interest due to the commercialization of chocolate for human consumption. Several studies provided evidence about the non-biological factors involved in its translocation into the cacao plant. However, factors related to this process, including soil bacterial community composition (SBCC), still need to be addressed. It is well known that soil microbiome could impact compounds’ chemical transformation, including Cd, on the field. Here, we found the first evidence of the link between soil bacterial community composition and Cd concentration in cacao soils and seeds. It highlights the importance of including the variation of bacterial communities to assess the factors driving the Cd translocation into cacao seeds. Moreover, the results highlight the relevance of the spatial heterogeneity within and across cacao farms, influencing the variability of Cd concentrations.

## INTRODUCTION

Cadmium (Cd) concentration is a quality-related issue in cocoa products (1) because cacao plants (*Theobroma cacao* L.) can accumulate heavy metals in their tissues (2–4). Cadmium reaches seeds and leaves through roots’ absorption of available cadmium from soil and its consequent translocation to plant tissues (5). This is an issue for cacao producer countries in the global south because the EU Commission Regulation of Food Safety recently imposed a regulation on Cd levels in different products like chocolates and derived products of cocoa (6), commonly exported from producer countries to EU (7). Raising safety standards from 0.1 to 0.8 mg kg^−1^ of Cd content in the final product (8, 9) is particularly important because of socioeconomic implications for local producers, mainly in developing countries. As a result, there has been a growing interest in factors driving Cd accumulation in cacao tissues (2, 4, 10–14).

Evidence suggests that Cd accumulation in cacao tissues is a multifactorial condition (2, 10, 11, 13–18), but the results are commonly partial or even contradictory in some cases. This may be a consequence of differences in studies focusing on contrasting factors (1), measuring Cd concentrations in different plant tissues (19), in different fractions with variable availability (i.e., soluble vs non-soluble [20]), or due to the variability of the sampling design (21). As proof of this, several studies have concluded that Cd content in plant tissues can be explained by factors as diverse as cation-exchange capacity, pH, texture/structure, organic matter content, electrical conductivity, aluminum content, interchangeable acidity, altitude, or landscape slope (13, 15, 18, 22–28). In addition, several studies have supported the idea that soil and seed Cd content is correlated (2, 12, 16–18, 28, 29) and linked to soil heterogeneity conditions (30), but others have shown precisely the opposite (13, 15). The last suggests that factors controlling Cd translocation from the soil to plant tissues are still under debate and deserve further analysis.

Regardless of the background of the study and the factors taken into account, the role of microorganisms as drivers of Cd mobility under field conditions is commonly disregarded and poorly understood (31–37). The last, despite the evidence showing that microbes have the potential to mediate the speciation of Cd through bioweathering (38, 39), the link between soil bacterial community dynamics and the mobility of Cd and other metals from the rhizosphere to the plants (23–25, 40–44) and, the initiatives to use them as key elements in cadmium bioremediation (39, 40, 45, 46). For example, microorganisms can drive the mobilization and root accessibility of metals by interacting with chemical chelators produced by plants (44) or directly by promoting the excretion of such exudates (47). Also, there is evidence that soil bacterial communities respond to substrates contaminated with Cd (48) and induce shifts in soil pH around the rhizosphere, increasing the solubilization of minerals and the mobility of Cd (49). However, most of these studies were performed under experimental conditions, overlooking the heterogeneity of the soil matrix and the diversity of microhabitats that exist in the rhizosphere (47, 50, 51), which in turn modulates the distribution and speciation of heavy metals in the soil (24, 52). Therefore, understanding the variability of Cd and microbial communities at a fine spatial resolution is fundamental to advancing our understanding of bacterial-mediated translocation of Cd from soil to plants and the spatial variation of Cd accumulation under actual cacao crop conditions.

To address this issue, we conducted a sampling design that included rhizosphere soil samples of 225 cacao plants distributed across five cacao farms under contrasting conditions in Colombia. We analyzed the soil bacterial taxonomic composition for each sample and measured soil Cd concentrations, physicochemical parameters, and Cd content in the corresponding fresh cacao seeds. This sampling design allowed us to analyze (i) the relationship between soil Cd concentrations and fresh cacao seed Cd content, (ii) the variation in soil and seed Cd concentration across and within farms, and (iii) its relationship with changes in soil bacterial community composition (SBCC). Our results provide evidence of the potential link between soil bacterial communities and the distribution of Cd across Colombian cacao crops, and highlight the importance of incorporating fine-spatial-scale studies to advance the understanding of factors driving Cd uptake and accumulation in cacao plants.

## MATERIALS AND METHODS

### Sample collection

The data used in this research were collected from cacao plantations in Colombia, which took place in Antioquia’s and Santander’s departments during 2020 and 2022. In the department of Antioquia, the cacao farms were located in two different geographical subregions, Urabá (8°37′8.19″ N and 76°36′23.80″ W, farms 1, 2, and 3) and Magdalena (6°49′30.93″ N and 73°27′2.17″ W, farm 5). In the department of Santander, the cacao farm was in the municipality of San Vicente de Chucurí (6°28′53.51″ N and 74°50′5.32″ W, farm 4). The last one has been evaluated in previous studies (30, 33), such as one of the farms with the highest soil Cd concentrations in Colombia. The plantations were selected to include heterogeneous conditions, such as cacao cultivars (phenotype), agroforestry arrangements, culture age, and agronomic management. The climatic conditions were classified as a tropical rainforest climate according to Köppens, with temperatures ranging from 26°C to 32°C and altitudes between 44 and 859 MAMSL (Table S1).

The criteria to establish the number of farms in each cacao department were (i) Antioquia sites with few recent reports about Cd in cacao crops (53), (ii) farmland selections with an expected cadmium gradient in soils (28, 53), and (iii) cacao-growing farms with contrasting Cd content and topographical conditions (30); this latest mainly referring to the hillside position of the cacao crop. For each plantation, a range of two to five sampling plots with characteristics such as proximity to the efflux irrigation water system, the transition between batches, or high-slope conditions were selected. The number of sampling plots within farms and plants within sampling plots was established considering the area planted with cacao trees and the representativeness and randomness of the plantation (30).

According to previous studies (54, 55), between 14 and 20 cacao plants were selected for each sampling plot within plantations. At the farms assessed, several agronomical conditions were found, one of them being the heterogeneity of the crop systems. Therefore, adding extra samples allows us to capture as much heterogeneity as possible. Besides, the extra samples were taken in case of losses of biological material during transportation from the field to the lab and due to bad quality during sequencing as a backup. Factors such as latitude, longitude, and elevation were registered using a GPS. We took samples of rhizosphere soil, leaves, and mature cacao pods from every plant selected. We considered the rhizosphere, the area adjacent to the roots, which we could visually corroborate. Three rhizosphere soil samples were collected from the top layer (20- to 30-cm soil depth) to avoid causing damage to the plants. Then, they were thoroughly mixed in the field into a composite homogenous sample per plant for an average Cd concentration measurement and bacterial community composition analysis. All samples were stored in Ziploc bags and were transported at a cooler temperature to the EAFIT University until they were processed for subsequent analysis. Soil samples were stored at −80°C before DNA extraction (54). Additional soil samples were sieved (2-mm mesh) and air-dried for several weeks for subsequent physicochemical soil analyses (56). Leaves and pod (seeds) samples were stored at cooler conditions until they were processed for Cd quantification.

### Physicochemical characterization of soil samples

From each sampling plot into every plantation, a rhizospheric soil sample of 100 g was taken near every plant selected. Then, we mixed them into a composite homogenous sample to complete the physicochemical analysis (*n* = 16). Soils, leaves, and seed samples were transported to the Soil Chemistry Laboratory at the Industrial University of Santander (UIS) for Cd quantification, and each composite soil sample was dried. Samples that could not be processed directly were placed in freezer conditions until there was room in the oven. The soil samples were characterized by the soil texture or particle size that was measured following the methodology proposed by Bouyoucous with a hydrometer (57). The soil pH was determined in a soil/water solution (relationship 1:1 wt/vol) using a pH electrode (58). The cation-exchange capacity was measured using the ammonium acetate method (59). In contrast, the proportions of carbon and phosphorus were measured according to Walkley and Black (60) and Bray II (61) methods, respectively. The exchangeable cations Ca^2+^-Mg^2+^-Na^2+^-K^+^ were measured with atomic adsorption spectrophotometry (AAS) using the ammonium acetate extraction method (62), while boron and sulfur were measured by colorimetry using monocalcium phosphate extraction and by turbidimetric using monocalcium phosphate extraction (63), respectively. Fe, Mn, Cu, and Zn were measured by AAS using a diethylenetriaminepentaacetic acid (DTPA) extraction (64), and the electric conductivity was measured using an electrometric method.

### Measuring the total and available cadmium content in soils, leaves, and fresh seeds from cacao plants

The collected soil, leaves, and seed samples were pretreated to calculate the cadmium content after humidity correction. All samples were heated at 70°C for 4 hours, and the soil samples were sieved. The analysis of total cadmium concentration in soil and fresh seed samples was performed using the EPA-3050 reported method flame atomic absorption spectrometry (FLAA) (65), based on digestion using (HNO_3_)-perchloric acid HClO_4_. The microwave extraction and graphite furnace were used for fresh seed (GTA 120), and the heating plate was used for soil samples (Schott-Model CK111). The highest seed Cd content samples were processed with a graphite furnace. After extraction, the Cd content was measured by flame atomic absorption spectrophotometry or AAS by direct aspiration (Agilent Technology model AA240FS) (66) using Cd(NO_3_)_2_ in HNO_3_ Suprapur 0.5 mol/L as reference material or standard. Total soil and fresh seed Cd concentration was measured for each plant (*n* = 225) and leaves. Cd concentration was measured per sampling plot (*n* = 16). The available cadmium concentration was measured in 80 samples (*n* = 80), selected according to what we observed in the total cadmium tendency of the samples. The metal extraction of available Cd was based on the DTPA method for this group of samples (67), and the measurement was made with AAS with a graphite furnace.

The translocation factor was calculated using both the total soil Cd concentration and the seed Cd content of each plant. The translocation factor is a method to calculate cadmium transfer from soil to plants’ aerial tissues. This parameter was calculated through the ratio of Cd present in plant tissues divided by the pseudo-total Cd found in the soil (68–70).

### Soil bacterial communities and DNA extraction

For bacterial community composition analysis, we used composite rhizosphere soil samples from each cacao plant (i.e., 225 samples collected overall). The samples were transported and stored at 4°C until they were processed. We sieved soil samples to remove rocks, invertebrates, or leaves as preparation for the DNA extraction process. According to the manufacturer’s instructions, DNA was extracted from 0.25 g of soil per sample using the DNeasy Powersoil Pro Kit (Qiagen, Germantown, MD, USA). The DNA concentration was assessed by gel electrophoresis at a concentration of 1.2% agarose and was measured by both the Nanodrop spectrophotometer (Thermo Fisher Scientific, USA) and the Qubit 3.0 Fluorometer (Thermo Fisher Scientific, USA) techniques. Then, the sequences were amplified using a polymerase chain reaction for rDNA 16S with the standard primers 515F/806R (42). The sequencing process was carried out using paired short-reads of 300 bp from the 16S V3-V4 region in an Illumina MiSeq platform (71) at the University of Massachusetts facility at Dartmouth, MA, USA. This study’s 16S rDNA gene sequences were stored in the National Center for Biotechnology Information Sequence Read Archive (PRJNA1015591).

### Bioinformatics analysis of bacterial community

A total of 225 bacterial community samples were analyzed using a filtered process by Qiime2 implementation 2019.4 (72). Raw data were quality checked with FastQC. The 16S rDNA amplicon sequence variants (ASVs) were inferred using the DADA2 package pipeline in the Qiime2 (72, 73). DADA2 filters read, denoises, merges paired-end reads, and removes chimeras. Forward and reverse reads were truncated at 230 and 200 bp, respectively, and sequences without the expected length were removed from the data set. Chloroplast, mitochondria, and Archaea sequences were eliminated from the data set to construct the ASV table for downstream analyses. We assigned the taxonomy to the SILVA reference database (v.132) for prokaryotes. Finally, the data were organized with phyloseq (Bioconductor v.3.15), where the species-level sequence identification was carried out with an identity of 100%. For all downstream analyses, we used rarefaction to 5,000 reads/sample as a normalization method. This is to prevent biases on the estimations of alpha and beta diversity metrics (74).

### Statistical analyses

The R software v4.1.0 (R Core Team 2021) was selected for all statistical analyses, including physicochemical data by principal components analysis (PCA), simple regressions, linear mixed-effects (LME) model, stepwise regression, bootstrap, and beta diversity based on the ASV’s table. Based on the Bray–Curtis and Euclidean distances, a Mantel test was performed to determine the correlations between bacterial communities and translocation factors in fresh cacao seeds. PERMANOVA results were obtained using Adonis2 (vegan::adonis2), and then similarities were analyzed to assess differences among study sites, cacao varieties, topographical conditions, and Cd concentrations.

The LME model and the stepwise regression model were used to identify the most critical parameters to seeds cacao Cd bioaccumulation, identifying soil physicochemical parameters with the most correlated factor in that process. Then, a bootstrap analysis was made to estimate the percentage of the variance explained by each predictor within each multiple regression model. Moreover, a Pearson correlation was conducted using cor: correlation, variance, and covariance (matrices) function in R (75). The Corrplot package (76) was used to visualize the correlation between physicochemical parameters and fresh seed Cd content.

Additionally, a non-metric multidimensional scaling (NMDS) analysis was carried out on the pairwise Bray–Curtis dissimilarities between soil bacterial community composition within and between farms, ensuring that stress values indicate a good fit. Then, we extracted the first ordinal axis to correlate it with fresh seed and total-available Cd concentration in soils.

## RESULTS

### Samples collected

Two hundred twenty-five (*n* = 225) individual soil, leaves, and pod samples were collected: 185 proceeding from four plantations in Antioquia’s department and 40 samples coming out from one plantation in the Santander department (Fig. S1).

### Variation of Cd concentrations across and within farms

**Fig 1:**
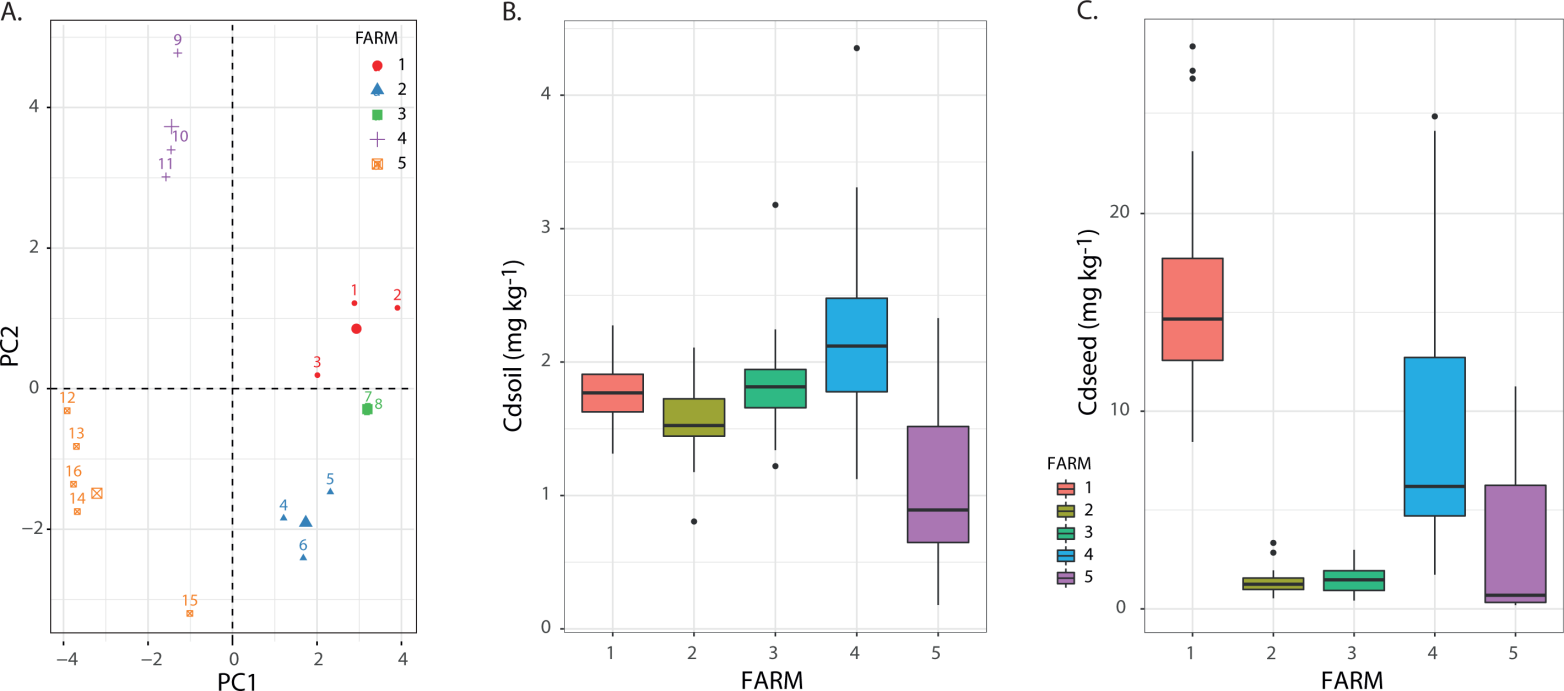
(A) PCA of sampling plot from each farm based on averaged Cd concentrations and other environmental variables measured on each plot. Colors and shapes represent a different farm. (B) Cdsoil concentration (mg kg-1) and (C) Cdseed content (mg kg-1) for each farm. Different colors represent different farms.

Our analysis captured a broad range of soil Cd concentration (Cd_soil_) and seed Cd content (Cd_seed_) that were associated with different environmental conditions measured on each plot (Fig. 1; see Fig. 4 for correlations). The results suggest that samples are grouped by farms according to their Cd concentrations in soils and seeds (Fig. 1A).

The Cd_soil_ ranges from 0.1 and 4.3 mg kg^−1^ (Fig. 1B). This variation is about 50% of the total range in soil Cd concentrations reported within a national survey of Cd across 1.837 cacao soils from Colombia (i.e., 0.01–27 mg kg^−1^) reported previously (30). Overall, farms 1 to 3 showed similar Cd_soil_; in contrast, farms 4 and 5 evidenced a higher dispersion (Fig. 1B). In addition, average Cd_soil_ was higher than 1 mg kg^−1^ in four out of five farms, a value used as a threshold by Finland, Belgium, and Austria for agricultural purposes (77). On the contrary, the variation in Cd_seed_ was higher than the topsoil Cd concentration, ranging from 0.4 and 28 mg kg^−1^ across farms. The highest values were detected in farms 1, 4, and 5, with consistently significant variations within each farm. In contrast, farms 2 and 3 were consistently lower and less variable than the rest (Fig. 1C).

### Exploring the relationship between soil Cd concentration and seed Cd content

**Fig 2:**
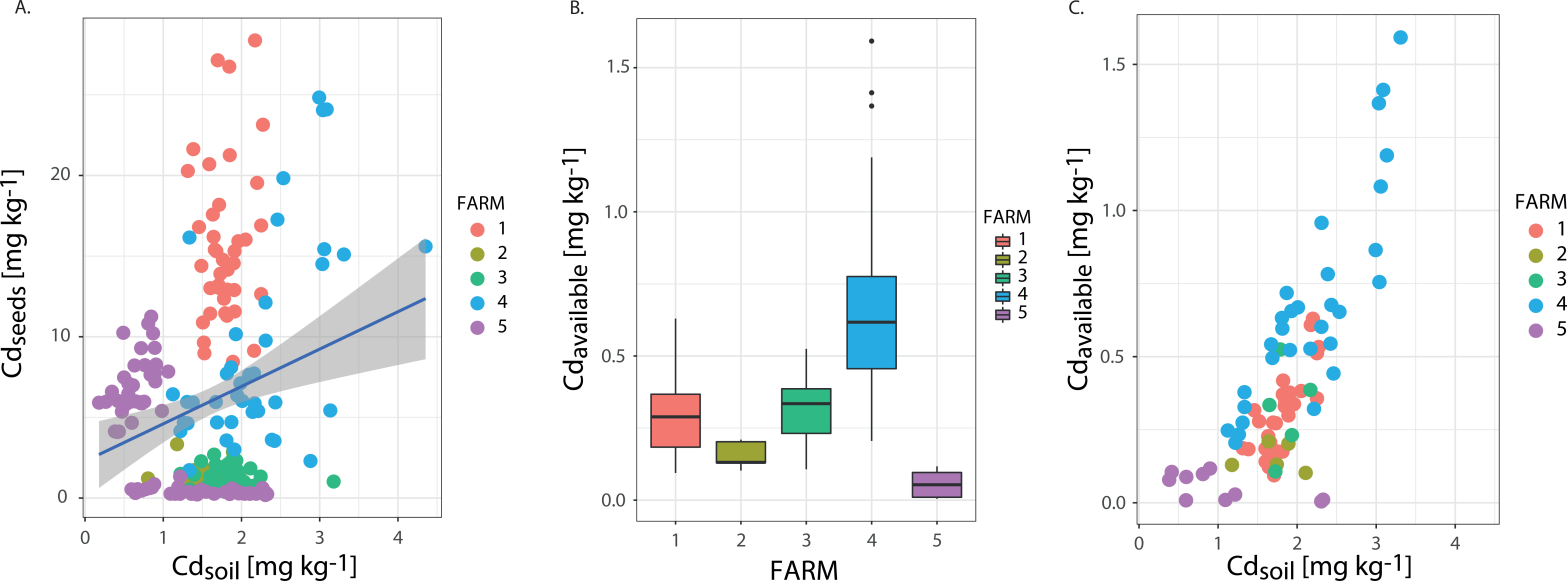
(A) Simple regression analysis of Cd_soil_ concentration (mg kg^−1^) and Cd_seed_ content (mg kg^−1^) in each farm. Different colors represent different farms, and every point corresponds with every sample collected. (B) Cd_Avail_ concentration in soils (mg kg^−1^) in each farmland. (C) Simple regression of Cd_Avail_ and Cd_soil_ concentration in soils (mg kg^−1^). Every color corresponds to different farms.

A descriptive exercise using a simple regression model showed a very weak, however, significative relationship between Cd_soil_ and Cd_seed_ across the samples (R = 0.21, *P* value < 0.01, R^2^: 0.045) (Fig. 2A). To test this further, we then controlled the variability between and within farms (i.e., between plots) using an LME analysis, by including farm and plot as random variables (78). The LME showed that the random effects variance for between-farms and within-farms models corresponds to about 70% and 72%, respectively (Table 1, models 1–3), so farm and plot explain a large portion of the variation between Cd_soil_ and Cd_seed_ (analysis of variance [ANOVA]_FARM_, *P* < 0.01; ANOVA_PLOT_, *P* < 0.01). Finally, the comparison of the Akaike information criterion (AIC) between these three models showed that model 3 is the one that fits the best Cd_soil_ and Cd_seed_ relationship (*P* < 0.01, AIC = 367.9, Table 1, model 3), suggesting that the total amount of Cd_soil_ cannot explain by itself the differences in Cd_seed_ between farms (model 1, *P* > 0.05, AIC = 444.2, Table 1). Instead, these results suggest that the variation within and between farms plays a role in explaining the Cd_soil_ and Cd_seed_ relationship.

**TABLE 1:** LME model implemented several combinations of Cd_seed_, Cd_soil_, and Cd_Avail_ according to locations of sampling design (FARM and FARM:PLOT)

Model number	LME model	AIC	Variance random effect	Variance residual random effect	Fixed effects Pr(>|t|)
1	lmer(Cd_seed_ ~ Cd_soil_ + (1|FARM), data = cadmium, REML = F)	444.2	0.7020	0.3688	0.993
2	lmer(Cd_seed_ ~ Cd_soil_ + (1|FARM:PLOT), data = cadmium, REML = F)	372.3	0.7156	0.2259	5.59e-8^*a*^
3	lmer(Cd_seed_ ~ Cd_soil_ + (1|FARM) + (1|FARM:PLOT), data = cadmium, REML = F)	367.9	0.7764	0.2261	3.87e-7^*a*^
4	lmer(Cd_seed_ ~ Cd_Avail_ + (1|FARM), data = cadmium, REML = F)	179.3	0.4310	0.4239	3.95e-5^*a*^
5	lmer(Cd_seed_ ~ Cd_Avail_ + (1|FARM:PLOT), data = cadmium, REML = F)	174.8	0.5131	0.3245	3.34e-7^*a*^
6	lmer(Cd_seed_ ~ Cd_Avail_ + (1|FARM) + (1|FARM:PLOT), data = cadmium, REML = F)	169.9	0.536	0.3204	4.99e-7^*a*^
7	lmer(Cd_soil_ ~ Cd_Avail_ + (1|FARM), data = cadmium, REML = F)	161.1	0.0357	0.3768	1.48e-11^*a*^
8	lmer(Cd_soil_ ~ Cd_Avail_ + (1|FARM:PLOT), data = cadmium, REML = F)	159.4	0.1796	0.3078	1.32e-13^*a*^
9	lmer(Cd_soil_ ~ Cd_Avail_ + (1|FARM) + (1|FARM:PLOT), data = cadmium, REML = F)	161.2	0.17708	0.30829	2.16e-12^*a*^
10	lmer(Cd_soil_ ~ SBCC + (1|FARM) + (1|FARM:PLOT), data = cadmium, REML = F)	444.4	0.54169	0.34617	2.19e-6^*a*^
11	lmer(Cd_seed_ ~ SBCC + (1|FARM), data = cadmium, REML = F)	414.0	0.6187	0.3442	6.74e-6^*a*^
12	lmer(Cd_Avail_ ~ SBCC + (1|FARM:PLOT), data = cadmium, REML = F)	−3.1	0.002323	0.048670	7.17e-9^*a*^

^*a*^ Corresponds to a fixed factor term significantly affecting the response with a *P-value* ≤ 0.001.

Furthermore, a subset of soil samples (*n* = 80) was selected to assess if Cd_soil_ is a good proxy of Cd_Avail._ Our analysis shows similar patterns of variation across and within farms (Fig. 2B vs Fig. 1B) and a relationship between Cd_soil_ and Cd_Avail_ (Fig. 2C). In fact, Cd_Avail_ represented between 9.3% and 27.2% of Cd_soil_ across farms. Finally, we found comparable results when using the LME modeling approach to replace Cd_Avail_ instead of Cd_soil_ (Table 1, models 4, 5, and 6). However, using Cd_Avail_ and controlling variation between farms (compare models 1 and 4), we observed that the cadmium available is a better predictor of Cd_seed_ than Cd_soil_.

### Assessing the role of soil bacteria on the variation of Cd_soil_ and Cd_seed_

**Fig 3:**
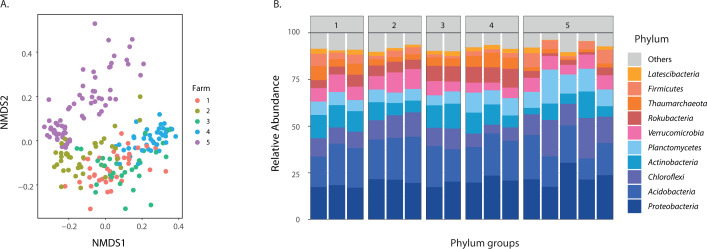
(A) NMDS of SBCC when every point corresponds to every sample collected in each farm (different colors). (B) Taxonomic assignment of relative abundance at the phylum level, covering around 90% of the total relative abundance, each number in boxes corresponds to the farms, each line into the boxes corresponds to sampling plots.

The NMDS was used to analyze the changes in Bray–Curtis dissimilarities between communities, summarizing the variation of SBCC along two synthetic axes (i.e., NMDS1 and NMDS2). The NMDS showed a clear segregation of bacterial communities between farms (*P* value < 0.001, PERMANOVA). Still, high variability and some overlap were observed within farms (Fig. 3A) Moreover, the taxonomic assignment of SBCC by farm showed that phyla such as Proteobacteria, Acidobacteria, Chloroflexi, and Actinobacteria represented between 50% and 65% of the total relative abundance (Fig. 3B). However, some differences were detected in some of the phyla with less relative abundance, such as Thaumarchaeota, Rokubacteria, and Firmicutes. A subsequent companion paper will explore the details of the bacterial community concerns in Cd_soil_(C. Jaramillo-Mazo, J.C. Alvarez, unpublished data).

**Fig 4:**
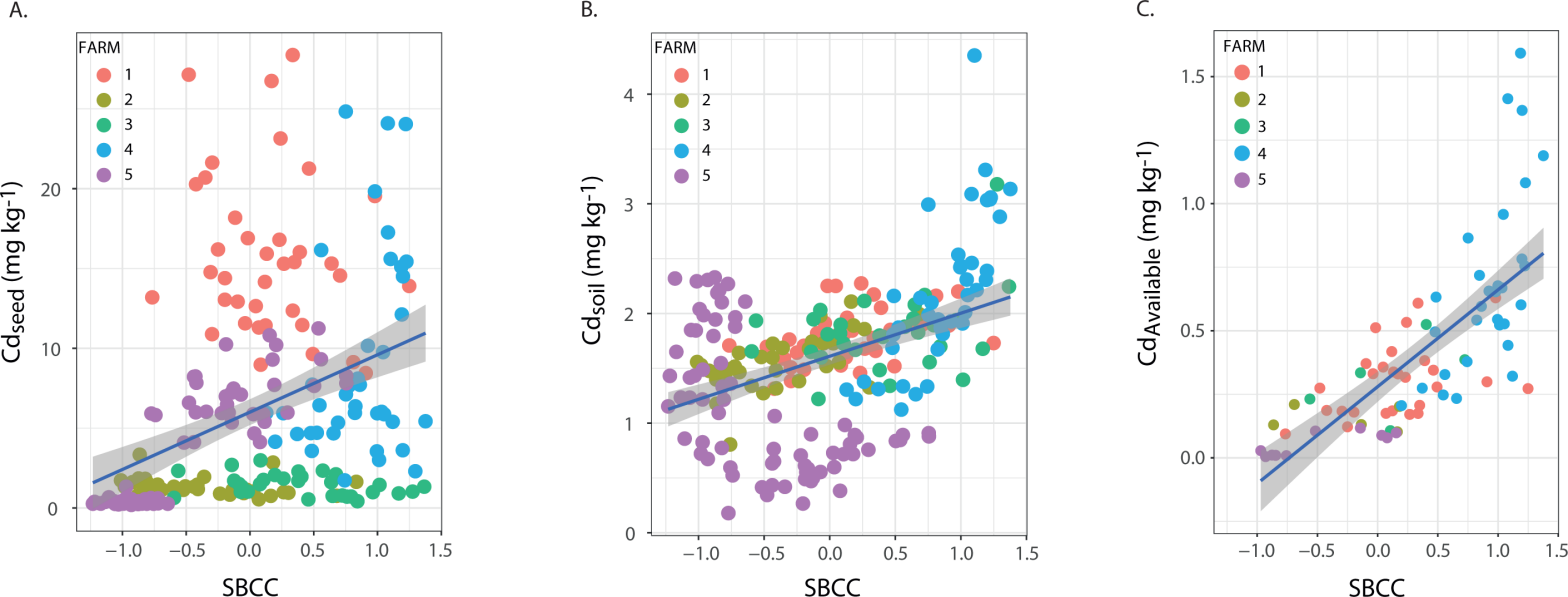
Simple regression of (A) SBCC and Cd_seed_ (mg kg^−1^), (B) SBCC and Cd_soil_ (mg kg^−1^) and, (C) SBCC and Cd_Avail_ (mg kg^−1^) in each farm. Each color represents a different farm.

The SBCC dissimilarities were related to Cd_seed,_ Cd_soil_, and Cd_Avail_ to explore the role of bacteria communities on Cd distribution across cacao crops. To do that, we extracted the NMDS axis coordinates, which summarize the variation in SBCC across all soil samples, so we had one coordinate per sampling point. Thus, coordinates that are closer imply similar communities, whereas coordinates that follow far apart imply different communities. We used the NMDS1 axis as an independent variable in a linear regression model, where Cd_seed_, Cd_soil_, and Cd_Avail_ were the dependent variables (Fig. 4A through C); we were able to test the relationship between bacterial community composition and Cd. Finally, the relationship between SBCC and Cd_seed_ was evaluated using a simple regression model and was validated with an LME analysis, which controls the variation across farms. Both regression models show that SBCC is related to Cd_seed_ (Fig. 4B; Table 1, model 11); however, LME suggests that spatial variation across farms should be considered to fully explain the distribution of Cd_seed_. This observation was coherent with the association with the Cd translocation factor translocation factor (TF) variation across farms, a ratio between Cd_seed_ and Cd_soil_ (Fig. S2). Also, with Mantel tests that showed significative changes in the relationship between SBCC and BAF between farms, only two out of five farms (farm 2, farm 5) showed a significant relationship (Table S2).

### How do physicochemical factors change across the farms?

**Fig 5:**
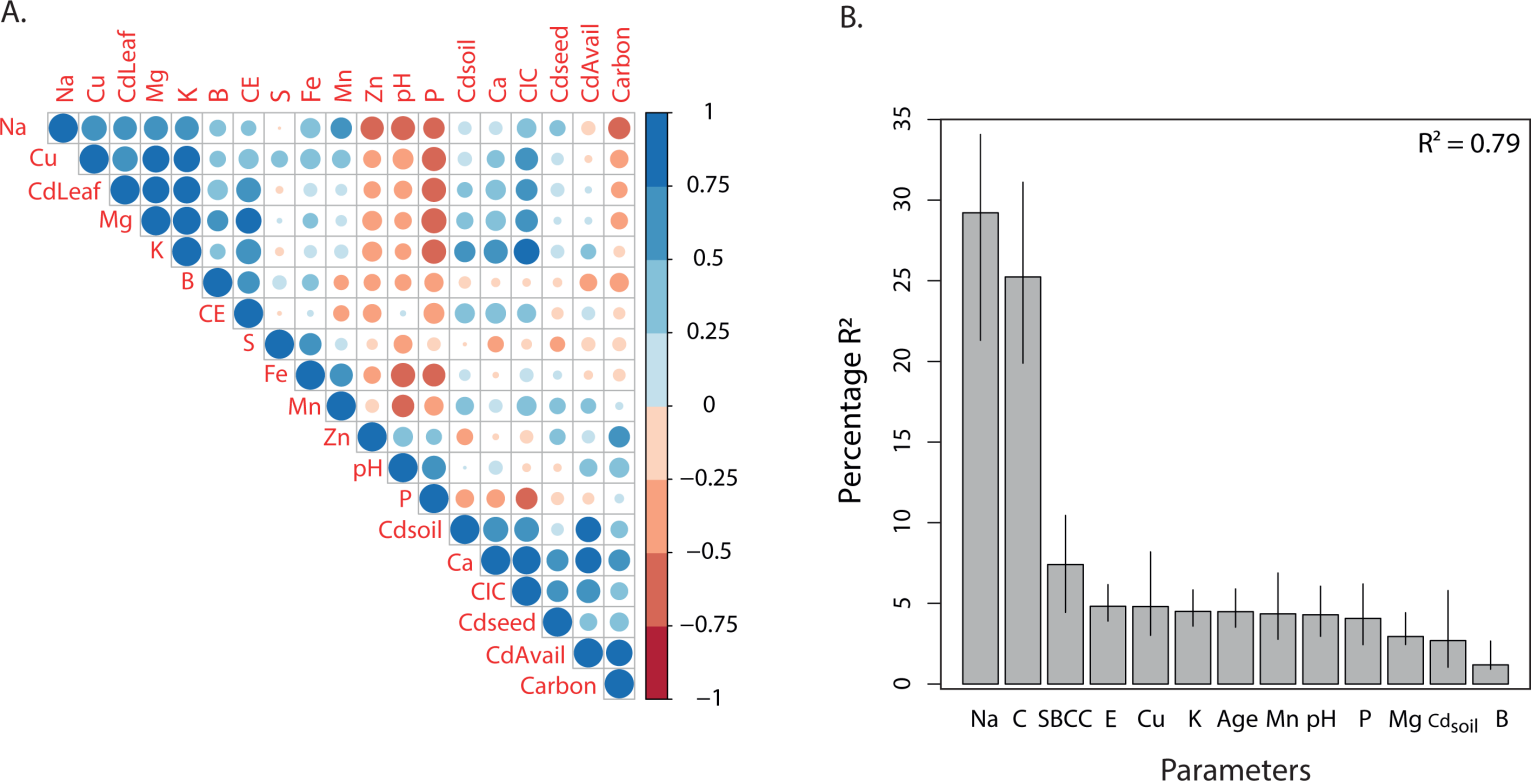
**(A**) Pearson correlation of physicochemical factors with Cd_soil_ concentration, Cd_seed_ content, and Cd_Leaf_ content. (**B**) Relative importance for Cd_seed_ with 95% bootstrap confidence intervals.

Farms were previously ordinated across a gradient of cadmium (Fig. 1). We used Pearson correlation analysis to study how different factors may be related to cadmium distribution across cacao farms (Figure 5). We found a positive correlation between Cd_seed_ and (i) Cd_Avail_, (ii) C content, (iii) Ca content, and (iv) cation-exchangeable capacity. At the same time, the leaves Cd content (Cd_Leaf_) was positively linked to Cu, P, Mg, Na, K, B, cation-exchangeable capacity, and electrical conductivity. Moreover, Cd_soil_ was associated with K, Ca, CIC, Cd_seed_, Cd_Avail_, electrical conductivity, and carbon content (Figure 5). These results were confirmed through a multiple linear regression by the stepwise and bootstrap methods (Fig. 5), which allowed us to decide which predictors were more important to explain each of the dependent variables. This selection was carried out according to AIC values (i.e., Akaike information criterion) generated by the stepwise procedure, which allowed us to compare the error and quality of each regression model, selecting the predictors with the lowest AIC values.

## DISCUSSION

*Theobroma cacao* L. can take up Cd from the soil and accumulate heavy metals in their tissues (11, 15, 21). Indeed, as previously mentioned, this condition reduces the commercialization of cocoa owing to the possible effect of Cd on human health (79, 80). Therefore, in this study, we measured the content of Cd in fresh seed, and we found that it shows an important variability both between and within farms under natural conditions, with an average of 3.49 mg kg^−1^ for the Magdalena River basin in Antioquia’s farmland (Fig. 1C, purple bar: farm 5). The values observed in this region include those obtained by Gil et al. (28) at the exact location. After checking for Cd content in the Urabá region in Antioquia (Fig. 1C, farms 1 to 3) and San Vicente de Chucurí in Santander (Fig. 1C, blue bar: farm 4), we observed a similar variability in cacao seed Cd content, ranging from 0.4 to 28 mg kg^−1^. These values were significantly higher than those reported for Colombia (28), Perú (11, 27), and Ecuador (18); however, they were similar to the range reported by Argüello et al. (17) in Ecuador. In three out of five farms, the Cd content in fresh seed exceeded the threshold limit (0.6 mg kg^−1^) (34).

Cd seed content in cacao crops is a multifactorial issue (19). In previous studies, this condition has been attributed to (i) soil physicochemical characteristics (15, 17, 18, 26–28), (ii) cacao cultivar type (2, 10), and (iii) soil total and available Cd concentration (10, 14, 17–19). However, studies on soil bacterial communities as a modulator of Cd accumulation and mobilization are only on its infancy. 19,1Although a few studies have measured multiple factors together (19), and the number of samples or parameters collected differed in some other reported studies (1), it seems pertinent to consider the concentration of Cd in soils to predict seed Cd content. Several studies have suggested a positive relationship between these conditions (14, 16–18). Recent studies have concluded that Cd_Avail_ in soils is a better predictor of Cd_seed_ in cacao crops (13, 19, 27) than total Cd_soil_. Our results suggest a similar effect, considering the AIC values obtained from LME modeling (Table 1, models 3 vs 6). However, the comparable spatial patterns obtained for both Cd_soil_ and Cd_Avail_ (Fig. 1B vs 2B and Fig. 2C) and the LME models (Table 1) pointed out the relevance of considering the small-scale variation to understand better the relationship between Cd concentrations in soil and seeds and including explicitly the variation within and between farms to capture soil heterogeneity (81). This result was confirmed by PERMANOVA analysis based on Cd_soil_ and Cd_Avail,_ which shows that samples coming from different farms (R^2^_FARM_ = *P* value *<* 0.01) and different plots within each plot (i.e., within farms, *P* value *<* 0.01, PERMANOVA, R^2^_farm_2_: 0.16, R^2^_farm_3_: 0.42, R^2^_farm_4_: 0.16, R^2^_farm_5_: 0.09) are significantly separated, although there is some degree of overlapping (Fig. 2A). Finally, we found a high variability in the ratio between Cd_soil_ and Cd_seed_ supports with particular disparities in farms 1 and 4 (Fig. 2A). Although this observation is coherent with the idea that Cd_soil_ is not the only factor explaining the Cd content in cacao crops, it cannot be attributed to a single factor. These differences may be attributed to factors as diverse as cacao plant varieties (2, 10, 13), the spatial resolution of the study (54, 82–84), or topographical conditions such as hillside position (85), but it clearly requires further analysis and research.

It is well known that soil bacterial communities change across ecosystem types (50, 86, 87) and heavy metal concentrations in agricultural farmlands (22, 23, 25, 43). However, one of the main achievements of this research was it provided new evidence of the association between soil bacterial community and Cd_seed_ content (Fig. 4A, *P* value < 0.01, R^2^: 0.15). Our results suggest that the Cd content observed in fresh cacao seeds may indirectly affect the soil Cd transformation mediated by the bacterial community variation (Fig. 4B, *P* value < 0.01, R^2^: 0.18). This is in agreement with other studies showing that Cd concentration in soils can generate shifts in total microbial biomass in bulk and rhizospheric soils (23, 24, 41, 43), which may be linked to Cd chemical species transformation (24, 25, 38–40, 42–44). Furthermore, we found that there is a significant and stronger relationship between SBCC and Cd_Avail_ (Fig. 4C, *P* value < 0.01, R^2^: 0.53), suggesting that bacteria may be involved in the chemical transformation of the heavy metals that modify the availability of these elements and its consequent translocation to plant tissue. Nonetheless, experimental evidence would be necessary to confirm this claim.

Other studies have described changes in the soil bacterial community under experimental setups with plants, such as rice, in soils contaminated with Cd (88, 89). These studies have revealed that regulating the composition of the rhizosphere bacterial community could simultaneously reduce Cd in rice grains (89). Although we could not offer such a functional link, we found a clear association between the coherently high variability in SBCC, Cd_soil_, and Cd_seed_ (Fig. 3B through D). In addition, one of the best LME models used to explain the variability of Cd_seed_ (Table 1, model 11) included SBCC as a predictor variable, only overcome by the models that included Cd_Avail_ or Cd_soil_ (Table 1, models 6 and 3, respectively). These LME results also confirm the relevance of the within-farm variation in analyzing these relationships; the last implies that the potential role of bacterial communities may vary from one agronomical unit to another.

In a previous work (7), the authors highlighted that in Ecuador (17) and Colombia (28), it was possible to predict cocoa bean Cd from soil parameters. Interestingly, the altitude of cacao farms was also an accurate predictor (R^2^ = 0.72) of Cd concentration in cacao seeds (28). Studies from Peru indicate similar findings (https://cacaodiversity.org/). In this study, the hills are recurrent even at the coastline of the Atlantic Sea, where the cacao farms in Necoclí, Antioquia, were assessed. The hillocks, or knolls, found in Necoclí had several altitudes. The knolls were lacking of gneiss and granite, as expected (90); however, depending on the fertilization strategy surveyed, the granite composition may vary on farms. Even though hills were constant, the altitude of the assessed farms varied from lower than 70 m in farm 1 to higher than 150 m in farms 2 and 3, respectively. On top of that, the size of the farms cropped with cacao and the agricultural management were seen as completely different, influencing the Cd content reported in cacao seeds in this study.

This study provides the first evidence of the link between changes in soil bacterial community composition and the distribution of Cd cacao soils and seeds, an important contribution to better understand the regulation of cadmium translocation to plant tissues. Moreover, the results direct our attention to the relevance of spatial heterogeneity within and across farms to assess the variability of Cd concentrations in cacao crops. Also, our data question the spatial resolution and sampling design commonly used in agronomic studies and the need of finer resolution to include microbe and heavy metal interactions in soils. Finally, the fact that the major shifts in taxonomic composition occur in low abundance groups like *Rokubacteria*, *Thaumarchaeota*, and *Firmicutes* (Fig. 3B) suggests that it is still unclear what portion of the bacterial community can respond to field Cd concentration gradients; an essential information to understand the actual extent of Cd as a selective factor on soil bacterial community composition.

## Data Availability

All the sequences obtained in this study are available in the NCBI SRA database at the BioProject accession number PRJNA1015591.

## AFTER EPUB

*[This article was published on 30 May 2024 with an error in the BioProject number in Materials and Methods. The number was corrected in the current version, posted on 4 June 2024.]*

## SUPPLEMENTAL MATERIAL

Supplemental material spectrum.03363-23-s0001.docx Fig. S1 to S4; Table S1 and S2.

ASM does not own the copyrights to Supplemental Material that may be linked to, or accessed through, an article. The authors have granted ASM a non-exclusive, world-wide license to publish the Supplemental Material files. Please contact the corresponding author directly for reuse.
