# Supplementary material for: Association between bacterial community and cadmium distribution across Colombian cacao crops
Source: Microbiol Spectr. 2024 May 30;12(7):e03363-23. doi: 10.1128/spectrum.03363-23 (PMC11218527; doi:10.1128/spectrum.03363-23)
Supplement: Supplemental material — Fig. S1 to S4; Table S1 and S2. [file spectrum.03363-23-s0001.docx]

**Supplementary Materials**


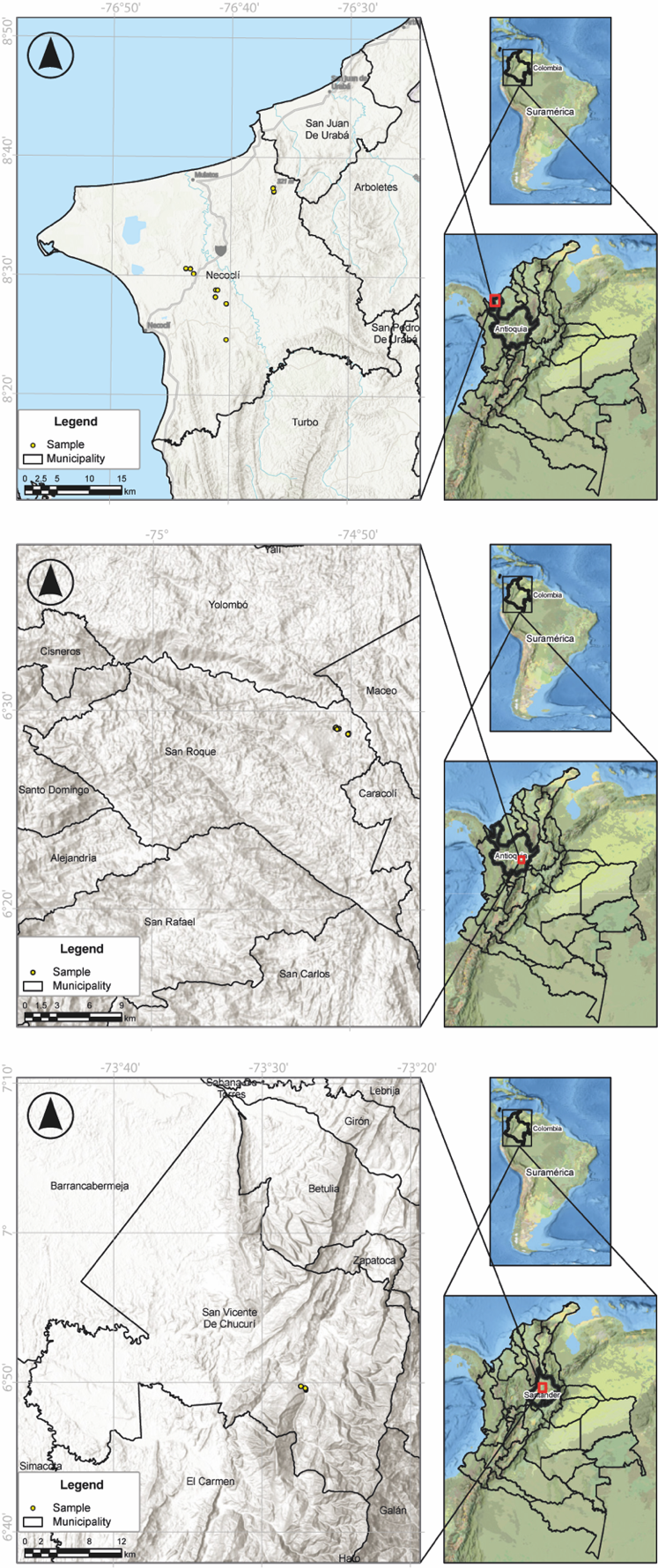


**Suppl. Fig. 1.** Scheme of departments where samples were collected: Antioquia and Santander.

**Suppl. Fig. 2.** Translocation Factor - BAF (ratio of Cd_seed_ divided by Cd_soil_) in every farm. Each color corresponds to a different farmland where sampling collection was done.

|  | ***Geographical Location*** | ***Cacao Varieties assessed*** | ***Weather/ mamsl*** | ***Age of crop*** | ***Agroforestry arrangement*** |
| --- | --- | --- | --- | --- | --- |
| *Farm 1* | 8°28'14.52" N 76°41'15.14"W | LK40  TSH565  FEAR5  ICS39 | 27.5°C  76 mamsl | 7 to 9 years ago | Wood tree species |
| *Farm 2* | 8°30'10.63" N 76°43'5.81" W | CCN51  LK40  CNCH13 | 28.3°C  65.2 mamsl | 5 to 7 years ago | Wood tree species |
| *Farm 3* | 8°37'7.49" N 76°36'23.79" W | ICS95  EET8  LK40 | 31.6°C  115 mamsl | 4 to 11 years ago | Avocado  Plantain |
| *Farm 4* | 6°49'37.10" N 73°27'5.87" W | CCN51  Hybrid varieties | 25.9°C  680 mamsl | 30 years ago | Avocado  Plantain  Citric crop |
| *Farm 5* | 6°29'07.8" N 74°50'36.2" W | ICS95  CCN51  ICS95  TCS01  TCS19 | 27.8°C  841 mamsl | 6 years ago | Wood tree species |

**Suppl. Table 1.** Cacao farm's location, environmental conditions, and crop characteristics.

| Mantel statistic based on Kendall´s rank correlation | | |
| --- | --- | --- |
|  | ***Mantel statistic R*** | ***Significance*** |
| Farm1 | 0.01423 | 0.405 |
| Farm2 | 0.1503 | 0.01 |
| Farm3 | 0.05704 | 0.161 |
| Farm4 | 0.05556 | 0.104 |
| Farm5 | 0.4213 | 0.001 |

**Suppl.Table 2.** Mantel statistic test based on Kendall´s rank correlation for every farm

**Suppl. Fig. 3.** LME modeling to samples per farm showing the relationship between Cd_soil_ and Cd_seed_. Each panel number corresponds to each farm; the points are the samples collected by location.

**Suppl. Fig. 4.** LME modeling to samples per sampling plot within farms showing the relationship between Cd_soil_ and Cd_seed_. Each panel number corresponds to each sampling plot; the points are the samples collected by location. Sampling plot 1 to 3 corresponds to Farm 1, sampling plot 4 to 6 corresponds to Farm 2, sampling plot 7 and 8 corresponds to Farm 3, sampling plot 9 to 11 corresponds to Farm 4, and sampling plot 12 to 16 corresponds to Farm 5.
